# Influence of incorrect staging of colorectal carcinoma on oncological outcome: are we playing safely?

**DOI:** 10.1007/s13304-021-01095-3

**Published:** 2021-07-06

**Authors:** Claudia Reali, Gabriele Bocca, Ian Lindsey, Oliver Jones, Chris Cunningham, Richard Guy, Bruce George, Stephen Boyce

**Affiliations:** 1 Department of Colorectal Surgery, Colchester General Hospital, Turner Road, 33 Groves Close, Colchester, CO4 5JL UK; 2 Department of Colorectal Surgery, Oxford University Hospitals, Old Road, Headington, Oxford, OX3 7LE UK

**Keywords:** Positive Margins, Adenocarcinoma, Colorectal Cancers, Stadiation, Outcomes

## Abstract

Accurate preoperative staging of colorectal cancers is critical in selecting patients for neoadjuvant therapy prior to resection. Inaccurate staging, particularly understaging, may lead to involved resection margins and poor oncological outcomes. Our aim is to determine preoperative imaging accuracy of colorectal cancers compared to histopathology and define the effect of inaccurate staging on patient selection for neoadjuvant treatment(NT). Staging and treatment were determined for patients undergoing colorectal resections for adenocarcinomas in a single tertiary centre(2016–2020). Data were obtained for 948 patients. The staging was correct for both T and N stage in 19.68% of colon cancer patients. T stage was under-staged in 18.58%. At resection, 23 patients (3.36%) had involved pathological margins; only 7 of which had been predicted by pre-operative staging. However, the staging was correct for both T and N stage in 53.85% of rectal cancer patients. *T* stage was understaged in 26.89%. Thirteen patients had involved(R1)margins; T4 had been accurately predicted in all of these cases. There was a general trend in understaging both the tumor and lymphonodal involvement (T *p* < 0.00001 N *p* < 0.00001) causing a failure in administrating NT in 0.1% of patients with colon tumor, but not with rectal cancer. Preoperative radiological staging tended to understage both colonic and rectal cancers. In colonic tumours this may lead to a misled opportunity to treat with neoadjuvant therapy, resulting in involved margins at resection.

## Introduction

Worldwide, colorectal cancer (CRC) is the third more frequent malignancy and the second most common oncological cause of death [1]. Accurate preoperative staging is vital in patients with colorectal cancer, as this highly influences their treatment [2–6]. Precise radiological assessment is critical in selecting patients for neoadjuvant therapy prior to resection. Inaccurate staging may lead to unexpected findings at the time of surgery, involved resection margins, and poor oncological outcomes. According to International Guidelines and literature, the radiological staging of colorectal malignancies relies on computed tomography (CT) with intravenous contrast (IV), CT Colonography, Magnetic Resonance Imaging (MRI), Endoscopic Ultra Sounds (EUS), and Positron Emission Tomography/Computed Tomography (PET) [7, 8]. Negative margins are mandatory to correctly manage the disease, and the incidence in elective settings is between 10 and 15% [9–15]. Positive resection margins are related to higher rates of recurrence and worse outcomes [13, 16–18]. Our aim is to determine preoperative imaging accuracy in colorectal cancer compared to histopathology and define the effect of inaccurate staging on patient selection for neoadjuvant treatment and positive resection margins.

## Materials and methods

All the data regarding patients with colorectal adenocarcinoma and undergoing a colorectal resection in a tertiary hospital, over a period of 4.3 years (from January 2016 to April 2020), were identified and collected on a prospective database. Demographic data, cancer and staging details, type of resection, neoadjuvant and adjuvant therapy were recorded. For each patient, the pTNM was compared with the cTNM to evaluate any under or over-staging. The last reported imaging after neoadjuvant therapy (ycTNM) was taken under consideration in the case of patients undergoing preoperative treatment. All the unmatched pTNM-cTNM were reviewed to determine if a correct staging could prevent the positive resection margin (PRM). The group of patients with PRM was compared with the group with negative margins to analyse possible affecting factors.

All the cancer cases were discussed in a multidisciplinary team (MDT) attended by the gastrointestinal radiologist, colorectal surgeons, oncologists, radiotherapists and gastroenterologists. The surgical procedures were performed by 5 experienced colorectal surgeons and the radiologic images were reported by 4 gastrointestinal radiologists. Indication for neoadjuvant and adjuvant treatment was given following the NICE guidelines.

All the colorectal resection for other causes (Inflammatory Bowel Disease, Lymphoma, Squamous Cell Carcinoma, Gastrointestinal Stromal Tumour) were excluded.

The observational study was carried out following the STROBE guidelines. The study was entered into the local audit register (ID 4927).

Univariable analysis was performed using ChiSquare Test for categorical data, whereas continuous data were analysed with Student’s T test or Mann–Whitney U test depending on the distribution. Logistic regression was used in the adjusted analysis for the relationship between variables for binomial data. Statistical significance was defined at *p* < 0.05. All statistical analysis was undertaken using R Studio Verison 3.1.1 (R Foundation, Boston, Massachusetts, USA).

## Results

Data were obtained from 948 patients (574male—60.54%) with a mean age at the operation of 59.31 (min32, max96) and mean BMI of 26.28 (min15.32, max41.71). Of those, 264 (27.85%) were affected by rectal cancers and 684 (72.15%) by colon tumours. Most of the procedures (83.43%) were carried out with a minimally invasive approach which included laparoscopy, robotic, and transanal Total Mesorectal Excision. The conversion rate was 10.74%, due to advanced tumor, obesity, dense adhesions, and bleeding. Only 49 patients (5.17%) underwent emergency operations because of obstructive or perforated cancers. Tumor location, type of resection, staging, and related treatment are summarised in Table 1, 2.

**Table 1:** Tumor location and Procedure information

Variable	Mean/*n*°	SD/%
Age*	59.31	21.69
BMI*	26.28	5.04
Male	574	60.54
Total R + ve	36	3.80
R1	31	3.27
R2	5	0.53
Minimally invasive procedures	791	83.43
Laparoscopic	733	77.32
TaTME	41	4.32
Robotic	17	1.79
Conversions	85	10.74
Emergency procedures	49	5.17
Procedures		
Right hemicolectomy	283	29.85
Extended right hemicolectomy	64	6.75
Resection of transvers colon	14	1.48
High anterior resection/left hemicolectomy	272	28.69
Low anterior resection	204	21.52
Abdominoperineal resection	57	6.01
Subtotal colectomy	20	2.11
Hartmann’s	34	3.59
Cancer location		
Appendix	6	0.63
Caecum	108	11.39
Ascending colon	144	15.19
Hepatic flexure	52	5.48
Transverse colon	50	5.28
Splenic flexure	17	1.79
Descending colon	52	5.48
Sigmoid/rectosigmoid colon	255	26.91
Rectum	208	21.94
Anorectal junction	56	5.91

*Mean values with SD

**Table 2:** pTNM and treatment information

Variable	Rectal Cancers *n* = 264		Colon Cancers *n* = 684		
	*n*°	SD%	*n*°	SD%	*p*
Lymph nodes harvested*	21.61	12.38	24.12	15.51	0.61
Neoadjuvant therapy	95	35.98	51	7.46	0.00001
Positive response	36	37.89	18	35.29	0.09
Adjuvant					
Administrated	86	32.57	237	34.65	0.54
Refused or unfit	22	8.33	72	10.53	0.31
pTNM					
T0-1	26	9.85	80	11.69	0.42
T2	74	28.03	98	14.33	0.00001
T3	147	55.68	351	51.31	0.23
T4	17	6.44	155	22.67	0.00001
N0	154	58.33	423	61.83	0.32
N1	85	32.20	171	25.00	0.06
N2	25	9.47	90	13.17	0.12
Distant metastasis	18	6.82	39	5.70	0.51
Lymphovascular invasion	92	34.85	156	22.81	0.0001
Minimally invasive resections	222	84.09	569	83.19	0.73
Conversions	20	9.01	65	11.42	0.32
Emergency procedures	0	0	49	5.17	0.00001

The mean number of lymph nodes harvested was similar for rectal and colon cancer (21.61 vs 24.12, *p* = 0.61). Statistically more patients with rectal cancers required neoadjuvant treatment (*p* < 0.00001) than those with colon tumours (35.98% vs 7.46%). However, adjuvant chemotherapy showed to be similar for both (*p* = 0.54).

Regarding the pTNM, rectal cancers were found to have more early cancers but less pT4 compared to colon tumours (*p* < 00,001), however, no difference was detected for the pathological lymph node staging and distant metastatic disease. The percentage of minimally invasive procedures and the rate of conversion was similar for rectal and colon resections (*p* = 0.73 and 0.32 respectively), but no emergency operations were performed for rectal cancers (*p* = 0.0001).

36 patients (3.80%) had a positive resection margin, 23 colon cancers (3.36%) and 13 rectal tumours (4.9%) [Table 3]. Only colonic resections had a specimen with R2 (5 cases) and they were procedures performed under emergency circumstances (10 operations). In both rectal and colon lesions, the circumferential resection margin was positive in most of the cases (84.64% and 86.96% respectively) and almost half of the R + ve colonic resections were in sigmoid cancers. All the positive resection margins (PRN) were in locally advanced cancer T3 or T4. However, T3 stage was significantly more represented in rectal cancers (*p* = 0.01), while T4 was in colon tumours (*p* = 0.01). No difference was shown for N staging instead. Most of the rectal resections with PRM were performed laparoscopically (*p* = 0.01) and those cases had a lower conversion rate (*p* = 0.02) in comparison to the overall resections. None of the colonic PRM underwent adjuvant treatment (*p* = 0.00001) because patients were deemed unfit or unwilling.

**Table 3:** Compared R1 resections in colon and rectal cancers

Variable	Colon R + ve		Rectal R + ve		*p*
	*n*°	%	*n*°	%	
R + ve	23	3.36	13	4.9	0.26
R2	5	21.74	0	0	0.33
R1	18	78.26	13	100	0.07
Margin involved					
Distal	2	8.69	1	7.69	0.91
Cimcunferential	20	6.96	11	84.62	0.84
Lymphnodal	1	4.35	1	7.69	0.67
Location					
Caecum	4	17.39	–	–	–
Ascending	5	21.74	–	–	–
Descending	3	13.04	–	–	–
Sigmoid	11	47.83	–	–	–
Staging pTNM					
T3	5	21.74	8	61.54	0.01
T4	18	78.26	5	38.46	0.01
N0	4	17.39	5	38.46	0.16
N1	9	39.13	2	15.38	0.14
N2	10	43.48	6	46.16	0.87
Minimally invasive procedures	9	39.13	11	84.61	0.01
Conversions	5	21.74	1	9.09	0.02
Emergency operations	10	43.48	0	0	0.01
Adjuvant therapy	0	0	12	92.31	0.00001

The overall TNM staging was correct in 28.79% of rectal tumours and 19.68% of colon cancers (*p* < 0.00001), but there was no statistical difference in the PRM cases (*p* = 0.75) [Table 4]. However, looking at the single T and N, colon cancers were better staged in both (*p* < 0.0001). In our sample, the stadiation tends to understage both T and N (*p* < 0.00001).

**Table 4:** Results comparing cTNM and pTNM

Variable	Rectal Cancers tot = 264		Colon Cancers tot = 635		
	*n*°	%	*n*°	%	*p*
Correct staging in R1	7/13	53.85	7/18	38.89	0.75
Correct TNM staging tot	76	28.79	125	19.68	0.00001
T					0.00001
Understaged	71	26.89	118	18.58	0.005
Overstaged	49	18.56	61	9.61	0.0002
N					0.00001
Understaged	115	43.56	129	20.31	0.00001
Overstaged	75	28.41	81	12.75	0.0001
Emergency procedures were excluded					

Reviewing all the cases, 5 patients with understaged colon cancer (0.79%), may have benefit of neoadjuvant treatment because of locally advanced disease (all elective cases). Of those, just 1 was a PRM (0.1%). However, none of the patients with rectal cancer had a possible compromised therapy due to incorrect stadiation.

Analysing possible factors that can contribute to a PRM in colon and rectal cancers, no connection was showed with age (*p* = 0.21, *p* = 0.34), BMI (*p* = 0.38, *p* = 0.09) and minimally invasive/open technique (*p* = 0.18, *p* = 0.10). However, there is a positive correlation between the PRM and height of the tumor form the anal verge (*p* = 0.009).

## Discussion

Nowadays, the staging of colorectal neoplasms is crucial for choosing the most appropriate treatment for each patient. Commonly, a MDT discussion should always be carried out at the moment of the diagnosis and periodically during therapies, to analyse patient’s features, diagnostic exams, and decide the best timing for oncological therapies and surgery. In this context, preoperative staging relies especially on radiological assessment, which helps the multidisciplinary team to plan the most suitable therapeutic pathway. We tried to assess how radiologic information can affect the operative outcomes and the decision to give preoperative treatment. In fact, despite we have many data in the literature assessing the accuracy of the different radiologic techniques, unfortunately, there is a lack of information that specify his impact on clinical decision during an MDT.

Our data showed that the radiological and clinical staging tend to underestimate the cancer TNM, but this affects clinical decisions and operative results in a very small amount of colon cancer cases. Furthermore, low rectal cancers seems to be more likely to have PRM possibly because of a more challenging operation.

According to international guidelines and literature, CT with IV contrast, MRI, EUS, and PET/CT are the mainstays of the staging of colorectal neoplasms.

Computed tomography with IV contrast is the first choice exam to stage colon cancers, as it gives precise information about the location and extension of the primary tumour, infiltration of nearby organs, and distant metastatic localisations. Nevertheless, it is not recommended for pelvic staging of rectal cancers, as it has important limitations in identifying bowel wall layers, its sensitivity and specificity in detecting local nodal disease (CT 55% and 74%, MRI 66% and 76% respectively) and CRM status are much lesser than those of MRI. Furthermore, CT ability to assess pelvic structures relation with tumour is poor [3, 6, 9]. Several studies have investigated the sensitivity and specificity of CT in staging colon cancer, and they fluctuate between 86–96% and 69–78% respectively (T1/T2 vs T3/T4). Significant differences can be highlighted between studies with slice thickness less or more than 5 mm (sensitivity 96% vs 71%, specificity 70% vs 73%). Correct T-staging ranges from 60 to 95% [10–12]. CT has noticeable limitations in detecting nodal disease, and its sensitivity and specificity vary between 6278% and 63–74% respectively, depending on the slice thickness. For N-status, correct staging is between 62 and 79% [11, 12]. CT with IV contrast shows reliable results in detecting secondary lesions. The sensitivity and specificity of CT to detect liver CRC metastasis is 85% and 97%. Only 25% of suspicious lung lesions are secondary, with an overall accuracy of around 83%. Overall, sensitivity and specificity for M staging were 85% and 98%, and statistics for correct M staging demonstrate relevant discrepancies, ranging from 55 to 100% [2, 3, 11].

Whilst MRI plays a secondary role in the preoperative staging of patients with colonic cancer, in rectal cancers it provides vital information on the local status and on its relations with pelvic structures (mesorectum, mesorectal fascia, CRM, EMVI, lymph nodes, intersphinteric plane, pelvic floor muscles, nearby organs) which are then crucial for treatment. According to International Guidelines and to the European Society of Gastrointestinal and Abdominal Radiology (ESGAR), MRI is compulsory and it is the technique of the first choice in staging and restaging rectal cancer, with the only exception of early malignancies, where EUS can best differentiate between T1 and T2 lesions [7, 8, 13]. In literature, the sensitivity and specificity of RMN in defining the T-status are around 85% and 77% respectively.

The N-status is a crucial prognostic factor for local recurrence, and the sensibility and specificity of MRI in identifying metastatic lymph nodes vary between 65–77% and 71–80% respectively.[3, 6, 14, 15]. Magnetic resonance imaging is the best technique to study the mesorectal fascia (MRF). Circumferential Resection Margin (CRM) is defined clear if > 1 mm from mesorectal facia, levator muscles and intersphinteric plane. By Contrast, threatened CRM is within 1 mm from MRF, or, if the malignancy involves the lower third of the rectum, within 1 mm from the levator muscles. The involvement of mesorectal fascia (MRF) is strongly linked to local recurrence, distant metastasis, and overall survival [16–19]. The MERCURY trial shows that the accuracy of MRI in predicting CRM before surgery was 88%. The sensitivity of RMI in defining CRM is between 59 and 94%, whilst its specificity oscillates between 85 and 98% [20]. The MERCURY trial 5 year follow up results show that CRM can be precisely defined preoperatively by MRI and categorise patients in low-risk and high-risk disease depending on the involvement of CRM. Patients with clear CRM had a 5-year overall survival of 62.2%, compared with 42.2% in patients with threatened circumferential margin [5].

The role of EUS is currently confined to the differentiation between T1 and T2 tumours, or if MRI is contraindicated [7, 8, 14]. Its sensitivity and specificity in staging T-status lay between 81 and 96% and 91 to 98%, depending on stage. EUS cannot reach high neoplasms, precisely determine cancer relations with pelvic structures in advanced bulky tumours, and its sensitivity and specificity in evaluating CRM involvement, EMVI, and mesorectal nodes (67% and 78%) are lower than those of MRI. Nodes outside the field of the probe and obstructive malignancies cannot be evaluated, and it is largely operator-dependent with an overall accuracy of 84% [2, 3, 6, 14, 21, 22].

PET/CT is confined to evaluation of equivocal findings at CT scan/MRI and/or absolute contraindication to IV contrast. Furthermore, its role in detecting nodal metastasis is limited by its low sensitivity (42.2%) and high specificity (87.9%), which make this exam less performant than CT and MRI [2, 3, 7, 8, 23, 24].

The residual tumour classification (R-TNM) is widely used as an important predictive feature. Surgical resection with a negative margin is a primary goal to achieve, to correctly manage the disease. The R classification is based on the histopathological presence or absence of residual tumour after the conclusion of the treatment: R0 no demonstrable residual tumour, R1 microscopic residual tumour, R2 macroscopic visible residual tumour. Residual tumour can also be discontinuous extensions of the primary (satellites, deposits, lymphatic and venous invasion, perineural invasion, and lymph node metastasis). Moreover, if distant metastasis is present, these also are coded as R + . Positive resection margins are highly related to increased rates of both local and systemic recurrence and outcomes as overall survival and disease-free survival are heavily affected [25–28]. According to the literature, the incidence of R1 resections in an elective setting in colorectal cancers is between 10 and 15% [20, 25, 29–34].

In our cohort, people affected by rectal cancer were found to have more pT2 and less pT4 than colonic cancers (*p* < 00,001), possibly because patients who underwent neoadjuvant CRT were more likely to be downstaged than those who had surgery as the first treatment. Actually, more rectal cancers patients had neoadjuvant treatment than those affected by colonic malignancies (35.98% vs 7.46%), *p* < 0.00001. Interestingly, data concerning surgical techniques indicates that a higher number of rectal resections were initially performed laparoscopically, and with lower rates of conversion to open surgery than colon resections. This may be due to a greater number of bulky cancers (T4) among colonic neoplasms, which could have persuaded the surgeons to perform directly either an open operation or an early conversion after the laparoscopic exploration. In our patients, preoperative cTNM staging was correctly given in more rectal cancer patients than those who were diagnosed with colon cancer, with statistical significance (*p* < 0.00001). A possible explanation might be that MRI is mandatory in the stadiation of rectal cancers, and this surely add significant information to the ones of CT scan. However, even though that group’s staging was more accurate, this did not influence the rate of R + ve, whose percentages were comparable in both samples (3.36 vs. 4.9%). In this study, if compared to final histopathology, figures show that there was a generalized inclination in downstaging preoperatively both T and N (*p* < 0.00001). Considering possible discrepancies in treatment related to an incorrect staging, (0.87% *n* = 5) patients diagnosed with colon cancer were initially downstaged, and their pTNM was higher than the expected preoperative cTNM. In these cases, a more accurate preoperative staging could have led the MDT through a different treatment choice, probably neoadjuvant therapy, to downstage the neoplasm. Nevertheless, of those five patients, just one had a R + ve resection. This means that inaccurate preoperative TNM staging was responsible of just one R1 resection, which represents the 0.1% of threatened margins which -perhaps- could have been prevented with neoadjuvant treatment. Conversely, no similar cases were observed in rectal cancers, possibly because MRI adds accuracy to their preoperative stadiation. In our sample, R1 resections were directly related to the distance from the anal verge: the lower the position of cancer, the higher was the probability to have a R + ve resection (*p* = 0.009). Furthermore, as far as R2 resections were concerned, they were all performed in patients affected by colonic cancers as well as emergency operations. The underlying reason may be that patients with colonic neoplasms were diagnosed with a higher number of bulky advanced malignancies (T4) than those affected by rectal cancers.

This study has some limitations. Firstly, data were collected retrospectively. Despite the high number of cases (nearly one thousand), the total number of R + ve was scarce. If we have had a higher number of R + ve resections, we could have obtained statistical significance. Moreover, the number of patients affected by colonic cancer was considerably higher than that of rectal cancer, and this could have been a confounding element.

Although our multidisciplinary team tried to strictly follow the NICE guidelines in giving indication to treatment for all colon and rectal cancers, other colorectal unit may have taken different decisions on some difficult cases. This may rain the issue on how generalisable our data could be. However, only a minority of cases could have led to a different decision in offering a neoadjuvant/adjuvant treatment, with a consequent small impact on the overall results.

## Conclusion

Preoperative radiological staging tended to understage both colonic and rectal cancers. In colonic tumours this may lead to a missed opportunity to treat with neoadjuvant therapy.
